# Transcription network construction for large-scale microarray datasets using a high-performance computing approach

**DOI:** 10.1186/1471-2164-9-S1-S5

**Published:** 2008-03-20

**Authors:** Mengxia (Michelle) Zhu, Qishi Wu

**Affiliations:** 1 Computer Science Department, Southern Illinois University, Carbondale, IL, 62901, USA; 2 Computer Science Department, University of Memphis, Memphis, TN, 38152, USA

## Abstract

**Background:**

The advance in high-throughput genomic technologies including microarrays has demonstrated the potential of generating a tremendous amount of gene expression data for the entire genome. Deciphering transcriptional networks that convey information on intracluster correlations and intercluster connections of genes is a crucial analysis task in the post-sequence era. Most of the existing analysis methods for genome-wide gene expression profiles consist of several steps that often require human involvement based on experiential knowledge that is generally difficult to acquire and formalize. Moreover, large-scale datasets typically incur prohibitively expensive computation overhead and thus result in a long experiment-analysis research cycle.

**Results:**

We propose a parallel computation-based random matrix theory approach to analyze the cross correlations of gene expression data in an entirely automatic and objective manner to eliminate the ambiguities and subjectivity inherent to human decisions. We apply the proposed approach to the publicly available human liver cancer data and yeast cycle data, and generate transcriptional networks that illustrate interacting functional modules. The experimental results conform accurately to those published in previous literatures.

**Conclusions:**

The correlations calculated from experimental measurements typically contain both “genuine” and “random” components. In the proposed approach, we remove the “random” component by testing the statistics of the eigenvalues of the correlation matrix against a “null hypothesis” — a truly random correlation matrix obtained from mutually uncorrelated expression data series. Our investigation into the components of deviating eigenvectors after varimax orthogonal rotation reveals distinct functional modules. The utilization of high performance computing resources including ScaLAPACK package, supercomputer and Linux PC cluster in our implementations and experiments significantly reduces the amount of computation time that is otherwise needed on a single workstation. More importantly, the large distributed shared memory and parallel computing power allow us to process genomic datasets of enormous sizes.

## Background

The rapid growth of genomic sequence data starting in early 1980's has spurred the development of computational tools for DNA sequence similarity searches, structural predictions, and functional predictions. The emergence of high-throughput genomic technologies in the late 1990's has enabled the analysis of higher order cellular processes based on genome-wide expression profiles such as oligonucleotide or cDNA microarray. A typical microarray dataset contains hundreds of sample points for thousands or tens of thousands of genes. A colossal amount of profound knowledge at genome level is hidden inside such immense expression data. A single gene is usually extracted to differentially identify expression genes at a significant level. However, such point level analysis does not address the full potential of genome-scale experiments. Nowadays genes can be affiliated by their co-regulated expression waveforms in addition to sequence similarity and proximity on the chromosome as in gene content analysis. Genes ascribed to the same cluster are usually responsible for a specific physiological process or belong to the same molecular complex. Such transcriptome (mRNAs) datasets deliver new knowledge and provide a revealing insight to the existing genome (genes) datasets, and can be used to guide proteome (proteins) and interactome research that aims to extract key biological features such as protein-protein interactions and subcellular localizations more accurately and efficiently.

However, organizing genome-wide gene expression data into meaningful function modules remains a great challenge. Many non-supervised and supervised computational techniques have been proposed to conjecture the cellular network based on microarray hybridization data. The widely employed techniques include Boolean network methods, differential equation-based network methods, Bayesian network methods, hierarchical clustering, K-means clustering, self-organizing map (SOM), and correlation-based association network methods.

Boolean network method is a coarse simplification of gene networks to determine the gene state as either 0 or 1 from the inputs of many other genes [1,2]. Differential equation-based network models gene networks as a set of nonlinear differential equations that indicate the gene rate change without the assumption of discrete time steps [3]. Bayesian network gives a graphical display of dependence structure based on conditional probabilities among genes. In hierarchical clustering, a dendogram is constructed by iteratively grouping together genes with the highest correlation, which is essentially a greedy algorithm achieving local optimality while disregarding negative association [4]. K-means clustering is an improved approach of hierarchical clustering requiring a subjective specification on the number of clusters [5]. SOM is a neural network-based iterative clustering method and also requires the user to estimate the initial cluster number [6]. The correlation-based association network technique has been commonly adopted to identify cellular networks due to its computational simplicity and the nature of microarray data (i.e., typically noisy, highly dimensional and significantly undersampled). However, the association network method relies on arbitrarily assigned thresholds for link cutoff, which inevitably introduces subjectivity in building network structure and topology. A novel technology, which can determine the structure of transcriptional networks and uncover biological regularities in a computerized and unbiased way, has been under active study by biological scientists.

There exists a wide range of microarray clustering and visualization tools available with statistical analysis support, including affy, cclust, cluster, mcluster, hybridHclust, SOM packages from R environment [7], integrated systems such as Bioconductor [8], and Cluster 3.0/Tree view [9], web-based systems such as cyberT [10], SNOMAD [11] and CARMAweb [12]. Many stand-alone systems are built upon R statistical packages using aforementioned clustering algorithms. We will discuss both the advantages and disadvantages associated with each clustering algorithm using real data examples in the next section. On the other hand, rapidly emerging large-scale genomic datasets pose a great challenge to current bioinformatics software and hardware resources. Most existing bioinformatics tools were developed as serial codes that are suitable for running on a single workstation, but often incur an unbearably long time delay or even cannot complete execution for large datasets due to limited memory. To date, cutting edge supercomputers such as IBM BlueGene, SGI Altix and Cray XT3, high-speed networks, high-performance storages as well as large-screen display devices have been in place or are being deployed across the nation. Efficient utilization of these high performance computing resources can help solve the problem of computation bottleneck and expedite the experimental turnaround time. The growing desire for improved application performance and reduced operational costs necessitates the design and development of parallel computing programs targeted at large-scale biological problems.

We propose to develop a system that constructs and analyzes various aspects of transcriptional networks based on random matrix theory (RMT) [13,14] using ScaLAPACK [15,16] for parallel calculation of linear algebra routines. We run our software package on two datasets: (i) yeast cycle microarray dataset [17] with about 2,500 genes and 79 samples, and (ii) human liver cancer microarray dataset [18] with about 20,000 genes and 207 samples. Comparisons are performed between the results generated by our package and some other popular packages.

## Results and discussion

The program in this work is implemented in C and MPI Fortran, and currently runs on a Linux cluster with eight nodes. We are now in the process of transiting our system from the Linux cluster to supercomputers with thousands of compute nodes. The experimental datasets are extracted from two public project websites, namely yeast cycle and human liver cancer projects.

### Yeast cycle dataset

Yeast cell cycling data is one of the best known microarray datasets that have been extensively evaluated. Since the structure of the network has been quite well understood, we are able to evaluate our clustering results by referring to an extensive set of published works.

#### Results from RMT method

The entire yeast genome is partitioned into a large number of functional modules sharing similar expression patterns. The large components of a deviating eigenvector computed from the Pearson correlation matrix are identified as gene members that belong to a specific functional module involved in a similar cellular pathway.

**Figure 1:**
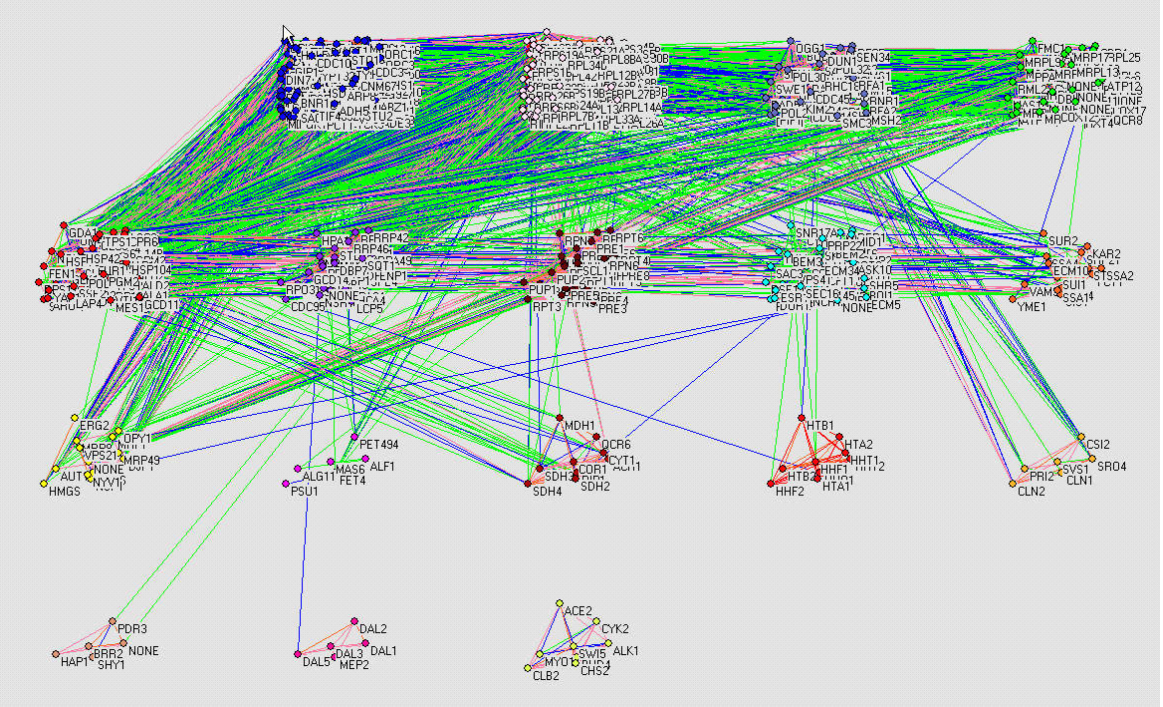
**Yeast cycle network**. Yeast transcriptional network labeled with gene names and created by pajek [25]

**Figure 2:**
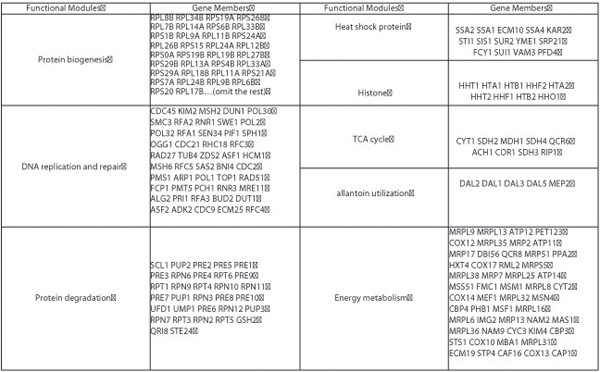
**Yeast cycle function modules**. Some functional modules and their gene members for yeast cycle genes

Fig. 1 and Fig. 2 show some distinct modules such as protein biogenesis, DNA replication and repair, energy metabolism, protein degradation, heat shock protein, TCA cycle, protein folding, allantoin mechanism, and histone. Various colors of the edges represent different ranges of correlation values between pairs of genes (vertices). By visual observation, we note that the correlations within groups represented by red or orange links are much higher than those between different groups represented by blue or green links, which strongly indicates the effectiveness of our clustering approach. For groups with a large number of genes, we recursively apply the same method to identify subgroups within large groups. Two major submodules for the first group with 230 genes in Fig. 1 are identified as glycolysis and cell cycle. By applying RMT method to the yeast cycle dataset, we have demonstrated that our results on functional module identification are consistent with available biological knowledge, which justifies the correctness of our approach.

#### Results from K-means clustering method

We use cclust package under the R environment to conduct K-means clustering, which repeatedly moves all cluster centers to the mean of their Voronoi sets. The distance between the cluster center and the data points is based on the Euclidian distance and polynomial learning rate is used. The major drawback of K-means method is that a user must specify the number of clusters, which is usually unknown for unexplored microarray datasets. For experimental purposes, we set the cluster number to be 20 based on previous results we obtained from the RMT method. K-means is able to identify protein biogenesis group with 114 genes; however some closely related protein biogenesis genes are assigned to several other unrelated groups. K-means algorithm tends to break down a coherent group with a large or medium number of gene members but lacks the capability of identifying small groups such as histone group of 9 genes, which has been successfully identified by our RMT method.

#### Results from hybrid hierarchical clustering method

Hierarchical clustering method has been widely used by contemporary biologists to cluster microarray datasets. Groups of genes are nested at different levels of details represented by a dendrogram. A user can choose either to build the hierarchical structure in a bottom-up or a top-down fashion. The bottom-up approach can identify small clusters but not large ones, while the top-down approach can easily discern a few large clusters. Chipman proposed a novel hybrid clustering method [19], which combines the advantages of bottom-up hierarchical clustering with that of top-down clustering. The key idea is to create mutual clusters comprised of members closer to each other than to any other members. A user can perform a constrained top-down clustering, which inhibits the breaking of any identified mutual clusters. We load the hybridHclust library into the R environment and run it on our yeast cycle data with 2,500 genes. Squared Euclidean distance is used to calculate the similarity between genes. Average linkage is exploited to join clusters. A user may also use single or complete for linking, which will only affect the dendrogram plotting not the mutual clusters. It generates a heavily cluttered dendrogram, which is tedious for users to interpret the nested structure even with a relatively small number of genes. When examining some of the mutual clusters, we find out that those mutual clusters are indeed highly correlated with each other. For example, five glycolysis genes are identified as one mutual cluster, eight out of nine histone genes are found within one mutual cluster. However, the size of a typical mutual cluster is generally quite small ranging from 2 to 8 with the majority of 2. We cannot use mutual clusters alone to identify any bigger size clusters. The mutual cluster method works well in recognizing distinct small size clusters. However, negative correlations providing important anti-regulation information in many cellular processes, are ignored in the similarity calculation. Moreover, mutual clustering is sensitive to small data variations which may easily cause gene membership change. Another problem associated with hybrid clustering is that with an increasing density of gene numbers, some genes will likely occur within the boundary of any mutual cluster, thus making it dificult to find mutual clusters [19].

### Human liver cancer dataset

We characterize the expression pattern of gene expressions in hepatocellular carcinoma (HCC) using RMT method. There are about 20000 genes with more than 200 samples, including 97 primary HCC, 76 nontumor liver tissues, 7 benigh liver tumor samples with 3 adenoma and 4 FNH, 7 metastatic cancers, and 9 HCC cell lines. We cluster the microarray data for both genes and samples. The liver samples are roughly divided into two major groups, namely the HCC tumor tissues and nontumor liver tissues, where a few HCC tumor samples are found in the nontumor cluster. Adenoma and FNH samples are dispersed within the HCC cluster. Metastatic colon cancer samples are identified as a single cluster due to their highly similar expression patterns. Two metastatic granulosa cell tumor samples are also grouped together. We observe that our method is also able to detect subclusters within a big cluster. For example, since tumor samples that are acquired from the same patient usually display similar expression patterns, 6 HCC samples from patient HK64 are grouped together as a subcluster within HCC cluster; 5 samples from patient HK66 are found in the same subcluster; 3 samples from patient SF34 also appear in one subcluster. Our clustering results conform nicely to the results published in the literature [20].

**Figure 3:**
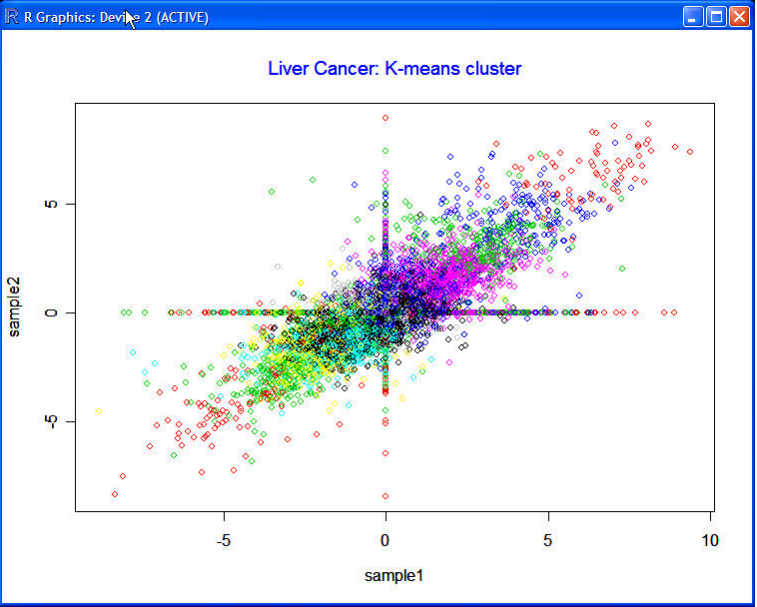
**K-means clustering results for human liver cancer genes**. K-means results with 20 clusters for human liver cancer genes

In addition to samples, we also successfully categorize the 20000 genes separately using parallel RMT program executed on an eight-node Linux cluster with a parallel computation time of about 20 minutes. However, domain knowledge in liver cancer is needed to elucidate our clustering results at a greater depth. The authors are seeking collaboration on this aspect. We also try to apply R hybridHclust library to the human liver cancer dataset. However, the hybridHclust library under the R environment cannot even read the raw dataset due to the upper limit on the loadable memory in Windows. K-means clustering is tested on human liver cancer data as well. Fig 3 illustrates the K-means clustering results of 20 different clusters. Each dot stands for one gene with different color representing different cluster membership. Different initial setting for the total cluster number produces significantly different clustering results, making K-means an unfavorable clustering approach for unknown microarray datasets.

## Conclusions

High-throughput genomic technologies such as microarrays have generated gene expression data at the transcription level. The unprecedented power for the study of gene expression of thousands of genes simultaneously can be potentially used to unveil the topology and functions of transcriptional networks. In this paper, we explored random matrix theory and parallel computing techniques to dissect transcriptional networks and identify various functional modules for large-scale microarray datasets.

Luo *et al*[21] also proposed a random matrix theory-based approach to infer transcriptional networks based on microarray data. However, their analysis is mainly focused on eigenvalues. In addition, their method require more computation cycles to calculate eigenvalues for many different correlation matrices. In our approach, we only need to compute eigenstates for one correlation matrix. We experimentally compare the performance between hierarchical clustering, K-means clustering, and our RMT method. Hierarchical clustering method is a very popular grouping technique used by biologists due to the simple underlying rationale and tree-like structure that can be easily visualized. However, similar to other heuristic approaches, it suffers from no guarantee of global optimization. Another problem with hierarchical clustering is that once a local grouping decision is made, no backtracking is possible. Moreover, when the total number of genes becomes prohibitively large, it is extremely dificult to analyze the nested tree structure to identify clusters. K-means algorithm, a typical partitioning based clustering method, seeks to find K clusters that minimize the sum of squared distances between each gene and its centroid. Input parameters such as the number of clusters and initial centroid locations need to be selected. However, different input parameters may lead to very different results, consequently leading to the problem of human subjectivity. K-means typically converges very fast, however, to a local optima, rather than the global optimum.

Our RMT method analyzes the genomic datasets from a global view. High levels of noise inherent to most biological datasets are removed first, and the true signal is further amplified for enhanced data interpretation. Consequently, RMT method avoids being trapped into local optima. Small-sized clusters that are easily mixed with other clusters by some clustering algorithms can be accurately extracted by our RMT method. Experimental results show that RMT method is able to recognize biologically meaningful clusters with various gene numbers ranging from two to several hundreds.

Most previous clustering methods partition members into non-overlapping groups. However, in our RMT method, one gene is allowed to reside in multiple groups, which supports a legitimate argument from the biological perspective that a single gene may get involved in different pathways. Transitively co-regulated genes, which are not directly correlated but both of which have correlation with the same gene, can also be detected and grouped. Our method is computationally efficient, objective without human intervention, and robust to high levels of noise. Functions of unknown genes are conjectured and explored through their associated function modules. This computational analysis is solely based on microarray data. If genes in the same functional module do not show a significant correlation, we will not be able to identify them using RMT method. However, it is likely that genes in the same functional module show a significant correlation under one condition but not under another condition, for example, the module of heat shock proteins is rarely identified in other yeast microarray datasets. By consolidating functional modules from multiple microarray datasets simultaneously, we will be able to improve the liability of the structure of functional modules. The authors will work toward this direction in the future.

## Methods

### Problem formulation

We define the expression signal of gene *i* = 1, …, *N* in various samples *s* = 1, …, *K* as:

(1) $$W_{i}\;(s)\;\equiv \;In\;\left(\frac{Es_{i}\;(s)}{Ec_{i}\;(s)}\right)\;,$$

where *Es_i_* (*s*) denotes the expression signal of samplesfor gene *i*, and *Ec_i_* (*s*) is the corresponding control signal. To account for various levels of expression signal shown by different genes, we normalize the data as:

(2) $$w_{i}\;(s)\;\equiv \;\frac{W_{i}\;(s)\;-\;\langle W_{i}\rangle }{\sigma_{i}}\;,$$

where $\sigma_{i}\equiv \;\sqrt{\langle W_{i}^{2}\rangle -\;{\langle W_{i}\rangle }^{2}}$ represents the standard deviation of *W_i_*, and *W_i_* stands for the average over different samples for gene *i*. From this normalized *N* × *K* data matrix *M*, we calculate the cross-correlation matrix *C* according to

(3) $$C\;\equiv \;\left(\frac{1}{K}\right)\;MM^{T}.$$

Pearson correlation coefficient *C_xy_* between gene *x* and *y*, each with *k* data series, can also be calculated from Eq. 4:

(4) $$C_{xy\;}=\;\frac{\sum \;(x_{i}\;-\;\bar{x})\;(y_{i}\;-\;\bar{y})}{(K\;-1)s_{x}\;s_{y}},$$

where *s_x_* and *s_y_* denotes the standard deviations. Pearson correlation ranges from 1 as perfect correlation to -1 as perfect anti-correlation. When *C_ij_* = 0, no correlation exists between genes *i* and *j*. However, conducting direct study on these empirical cross-correlation coefficients is rather difficult due to the unique properties of microarray experiments. Firstly, the cross-correlation between any pair of genes may not be constant: such co-regulations can fluctuate over time or under different sample conditions. Secondly, the limited number of samples that a microarray is typically conducted upon, may introduce significant “measurement noise” that compromises the accuracy of the underlying correlations. In order to filter out randomness contained within the empirical cross-correlation matrix, we test the eigenstates of this correlation matrix against those of a controlled counterpart, a truly random correlation matrix generated by computer random generator. Statistic properties that conform to the truly random matrix are labeled as noise contributions; on the other hand, any deviating eigenstates are treated as genuine correlations, which will be amplified and analyzed for transcriptional network construction.

### Deviating eigenvalues of the correlation matrix

**Figure 4:**
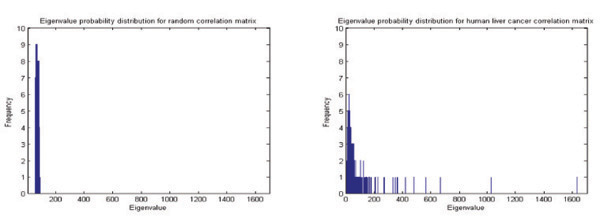
**Eigenvalue probability distribution**. Comparison of probability distributions for eigenvalues. Left: Eigenvalues calculated from a random correlation matrix *R*. Right: Eigenvalues calculated correlation matrix *C* from human liver cancer dataset.

We further compare the probability distributions *P^C^* (λ) and *P^R^* (λ) of the eigenvalues λ_*i*_ calculated from the cross-correlation matrix *C* and the random matrix *R*, respectively. Eigenvalues are arranged in an ascending order such that λ_*i*_ < λ_*i*+1_. The probability distributions *P^C^* (λ) and *P^R^* (λ) for human liver cancer data are plotted in Fig. 4. It has been observed that a set of the eigenvalues of *C* fall within the well-defined range of [λ_−_, λ_+_] calculated from *R*, with a few deviating from the upper (λ_+_) and lower bounds (λ_−_) conveying the true correlation information. This observation enables us to separate the real correlation from the randomness. This denoising process is necessary since microarray data is extremely undersampled and may introduce significant measurement noise. Interestingly, Kwapien *et al.*[22] found that increasing the length of time series or number of samples would cause eigenvalues to deviate more from the random matrix eigenvalue bounds. They declared that the bulk of the correlation matrix is not pure noise as conventionally thought to be. It could be possible that more subtle and less prevalent co-regulated gene groups could be squeezed out of the noise segment if we are able to acquire a larger sample size *K*. However, in practice, a large sample size *K* from the perspective of mathematical view is not always feasible for most biological datasets due to the considerable time and material resources involved in bio-related experiments.

### The components of the corresponding deviating eigenvectors

We consider the set of eigenvalues that deviate from the eigenvalue range of the random matrix as genuine correlation. The amount of variance contributed by each eigenvector (factor) can be reflected by the proportion of eigenvalue over the sum of all eigenvalues based on principle component analysis (PCA). In other words, principle factors are responsible for the majority of variation within the data. Thus, only large eigenvalues and their corresponding eigenvectors are retained for further treatment and gene group analysis. The rest of the eigenstates contain either insignificant or noisy information.

Deviating eigenvalues naturally lead us to the investigation of their corresponding deviating eigenvectors. There are *N* eigenvectors *u^i^* in total, *i* = 1…*N*. Each eigenvector *u^i^* has *N* components corresponding to *N* gene variables. All eigenvectors are perpendicular(orthogonal) to each other and are normalized to length of 1. The probability distribution of eigenvector components for different eigenvalues are plotted and compared against that of a random matrix, which follows Gaussian distribution with zero mean and unit variance.

(5) $$\beta \;(u)\;=\;\frac{1}{\sqrt{2\pi }}\;\exp\;\left(\frac{-u^{2}}{2}\right).$$

**Figure 5:**
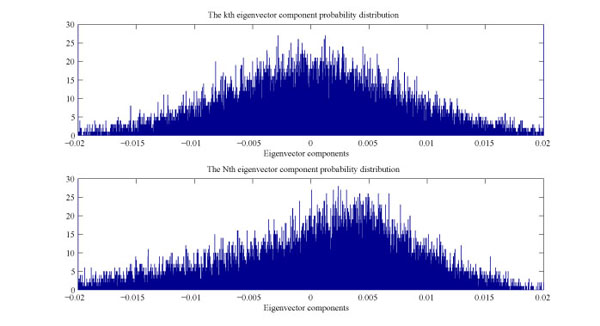
**Eigenvector components probability distribution for human liver cancer data**. upper: eigenvector components for *u^K^*, an undeviating eigenvector; lower: eigenvector components for *u^N^*, a deviating eigenvector.

The probability distribution of the eigenvector components with the corresponding eigenvalue λ_*k*_ from the bulk λ_−_ ≤ λ_*k*_ ≤ λ_+_ from human liver cancer data shows a good agreement with Gaussian distribution as indicated by the upper figure in Fig. 5. The deviating eigenvector components demonstrate a significant deviation from the Gaussian distribution as shown by the lower figure in Fig. 5. It has been also observed that the distribution curve is gradually reforming to approximate the shape of a Gaussian distribution when eigenstates approach the characteristic region represented by a random matrix.

### Loading factor and orthogonal rotation

After acquiring a set of normalized eigenvectors, we transform the eigenvector components to loading factors by taking the multiplication of vector components and the square root of corresponding eigenvalue. Each eigenvector represents one factor leading to one gene cluster. A larger loading factor (we select 0.5 to be the cutoff point) indicates that the corresponding gene “load” more on that eigenvector, or that gene is more expression-dominating for that cluster. To simplify the eigenvector structure and make the interpretation of gene clusters easier and more reliable, we apply orthogonal rotation to the retained eigenvectors. Since the rotation is performed in the subspace of the entire eigenstates, the total variation explained by the newly rotated factors are always less than the original space, but the variation within the subspace remains the same before and after rotation, only the partition of variation changes.

VARIMAX [23] is a simple and popular rotation method that transforms the principle data axes such that each eigenvector will contain a small number of large loadings and a large number of zeroes or small loadings. Biologically speaking, each gene tends to load heavily on only one or a few gene clusters. Thus, gene clusters consist of a reduced number of genes compared with pre-rotation results. The rationale behind VARIMAX is that a rotation (linear combination) of the original factors is searched in order to maximize the variance within factor loadings. A rotation matrix *R* can be determined to specify such rotation as following:

$$R\;=\;\left[\begin{array}{ll} \cos\;\;\theta_{i,\;i} & \cos\;\;\theta_{i,\;j}\\ \cos\;\;\theta_{j,\;i} & \cos\;\;\theta_{j,\;j} \end{array}\right],$$

where θ_*i,j*_ is the rotation angle from old axis *i* to new axis *j*.

### Stability of gene clusters in samples

The eigensignal *z^i^* (*s*) of a certain eigenvector *u^i^* under a sample series *s* is computed as the scalar product of the sample series on the eigenvector as shown in Eq. 6. The stability of gene clustering based on our eigenstates analysis can be evaluated in terms of variance of total expression signals denoted by *z^i^* for eigenvector *i* among different samples and time series. The variances are directly associated with the corresponding eigenvalues as one of the most important properties of eigensignals [22] in Eq. 7. The gene cluster derived from eigenvector with larger eigenvalue is more unstable compared with gene cluster associated with smaller eigenvalue. Note that variance levels indicate the consistence of gene members across different samples. Stability evaluation does not affect our clustering results. It only provides additional information on the quality of cluster membership.

(6) $$z^{i}\;(s)\;\;\equiv \;\sum_{k\;=\;1}^{N}u_{k}^{i}\;W_{k}\;(s)\;$$

(7) $$Stab\;\;(u^{i})\;=\;\sigma^{2}\;(z^{i})\;=\;{\left(u^{i}\right)}^{T}\;Cu^{i}\;=\;\lambda_{k}\;where\;\;i\;=\;1,...\;N$$

### Time-lagging expression behavior among genes

Time-series gene expression data provides temporal dimension of knowledge space. However, most similarity measurement techniques including Pearson correlation usually consider the expression patterns between two genes under the same conditions or at the same time points, no time-lagging analysis is taken into account. Thus, expression time-lagged genes will not be correctly identified as the same group. In fact, a certain time lag usually exists before a transcription factor begins to influence the expression of some other genes because of the delay in signaling mechanism. Such co-regulation behavior could be categorized as either up-regulation or down-regulation, namely the expression of a gene may either stimulate or inhibit the expression of other genes. It is our interest to capture and explore the time-lagging relationship among all the genes in our future work. Cross-correlation method [24] in Eq.8 calculates the correlation between two genes by taking the time-lagging into consideration.

(8) $$r_{xy}\;(k)\;\;=\;\frac{\sum_{t\;=1}^{N-k}\left(x_{t}\;-\;\bar{x})\;(yk\;+\;t\;-\bar{y}\right)\;}{\sqrt{\sum_{t\;=1}^{N-k}{\left(x_{t}\;-\;\bar{x}\right)}^{2}\;\;}\;\sqrt{\sum_{t\;=1}^{N-k}{\left(yk\;+\;t\;-\;\bar{y}\right)}^{2}\;}\;}$$

where *x_t_* is the expression signal of a known gene at time *t*, and $\bar{x}$ is the mean of *x_t_*. *y_k+t_* is the expression level of a gene at time *k+t*, and $\bar{y}$ is the mean of *y_y+t_*. *N* denotes the number of time points and *k* represents the time delay/lag. Likewise, the correlation will also be large if the first expression signal leads the second with the expression waveform shifted to the left of the second.

To capture the discrete correlation of two gene samples with time-series expression data, we can utilize discrete Fourier transform to compute the correlation. *Corr*(*x, y*)_*k*_ denotes the correlation of two genes *x* and *y*.

(9) $$Corr\;{\left(x,\;\;y\right)}_{k}\;\Leftrightarrow \;X\;(f)\;Y\;{\left(f\right)}^{*}$$

where *X*(*f*) and *Y*(*f*) are the discrete Fourier transforms of *x_t_* and *y_t_*, and the asterisk represents complex conjugation.

In addition, after genes are clustered by time-lagging analysis, people usually conduct upstream sequence analysis to identify consensus regulatory elements for those genes that are controlled by the same transcription factor.

### Parallel linear package ScaLAPACK

A single workstation is no longer fast and powerful enough to cope with emerging large scale microarray datasets. High performance computing facilities become indispensable tools for biologists to reduce the computing time and improve efficiency. In order to calculate the eigenvalues and eigenvectors for a large correlation matrix, we install ScaLAPACK on our Linux cluster. ScaLAPACK is an acronym for Scalable Linear Algebra Package, and is a library of high-performance linear algebra routines for distributed memory message-passing computers. PDSYEVX routine is used to compute selected eigenvalues and eigenvectors of a real symmetric matrix by calling the recommended sequence of ScaLAPACK routines. We use 2D block-cyclic data distribution for work load balance among all computer nodes to achieve performance and scalability. The size of the subblock dividing the symmetric correlation matrix is chosen to be 64 × 64, and the computer grid configuration is set to be 2×4 for an eight node cluster. With the help of parallel computing packages, we are able to finish some heavy computing tasks within short period of time (one hour), which might take up to days for a single workstation to run.

## Competing interests

The authors declare that they have no competing interests.

## Authors contributions

MZ designs and implements the RMT method on a single workstation. QW implements the parallel version of the RMT method. MZ also analyzes the yeast cycle and human liver cancer microarray datasets. Competing interests
